# Successful natural interferon-β plus ribavirin therapy in a chronic hepatitis C patient after discontinuation of interferon-α treatment due to arrhythmia and interstitial pneumonia

**DOI:** 10.1007/s12328-014-0500-8

**Published:** 2014-06-06

**Authors:** Akira Sato, Masahiro Yamauchi, Takayuki Yamada, Reiko Kumano, Kayo Adachi, Toshiya Ishii, Mikihito Hayashi, Daisuke Kumon

**Affiliations:** 1 Division of Gastroenterology, Department of Internal Medicine, St. Marianna University School of Medicine Yokohama City Seibu Hospital, 1197-1 Yasashicho, Asahi-ku, Yokohama, 241-0811 Japan; 2 Division of Cardiology, Department of Internal Medicine, St. Marianna University School of Medicine Yokohama City Seibu Hospital, 1197-1 Yasashicho, Asahi-ku, Yokohama, 241-0811 Japan; 3 Department of Radiology, St. Marianna University School of Medicine Yokohama City Seibu Hospital, 1197-1 Yasashicho, Asahi-ku, Yokohama, 241-0811 Japan

**Keywords:** Hepatitis C, Interferon-β, Interstitial pneumonia, Arrhythmia, Adverse effect

## Abstract

A 71-year-old female patient with hepatitis C virus genotype 1 had previously discontinued interferon (IFN)-α plus ribavirin therapy, pegylated IFN-α (pegIFN-α) monotherapy, and natural IFN-α monotherapy because of arrhythmia, interstitial pneumonia, and severe neurovegetative symptoms. She subsequently completed 72 weeks of natural IFN-β plus ribavirin therapy without remarkable adverse effects and achieved a sustained viral response, suggesting differences in the pharmacological properties and biological effects of IFN-α and IFN-β. Thus, natural IFN-β plus ribavirin therapy may be a treatment option for patients with poor tolerance to IFN-α or pegIFN-α treatments.

## Introduction

Treatments that help eradicate chronic hepatitis C virus (HCV) infections are essential for preventing disease progression to cirrhosis and hepatocellular carcinoma [1]. Interferon (IFN) is the standard therapy for chronic hepatitis C, and the combination of pegylated interferon-α (pegIFN-α) and ribavirin (RBV) therapy produces a sustained anti-viral response (SVR) in 50 % of patients infected with high viral loads of HCV genotype 1. Furthermore, higher SVR rates follow triple therapy with pegIFN-α and RBV plus telaprevir or simeprevir [2]. However, some patients fail to complete IFN treatments because of adverse effects such as interstitial pneumonia (IP), which is known as a critical adverse effect and can lead to death [3], and cardiovascular events such as arrhythmia, which are rare among IFN-treated hepatitis C patients [4]. In contrast, the rates of treatment discontinuation and/or dose modification due to severe adverse effects of natural IFN-β (nIFN-β) are reportedly low [5, 6], and the efficacy of combination therapy with RBV for genotype 1 chronic hepatitis C has been demonstrated [6].

Herein, we report successful nIFN-β plus RBV therapy in a chronic hepatitis C patient after discontinuation of IFN-α plus RBV therapy, pegIFN-α therapy, and natural IFN-α therapy following arrhythmia, IP, and severe neurovegetative symptoms, respectively.

## Case report

**Fig. 1:**
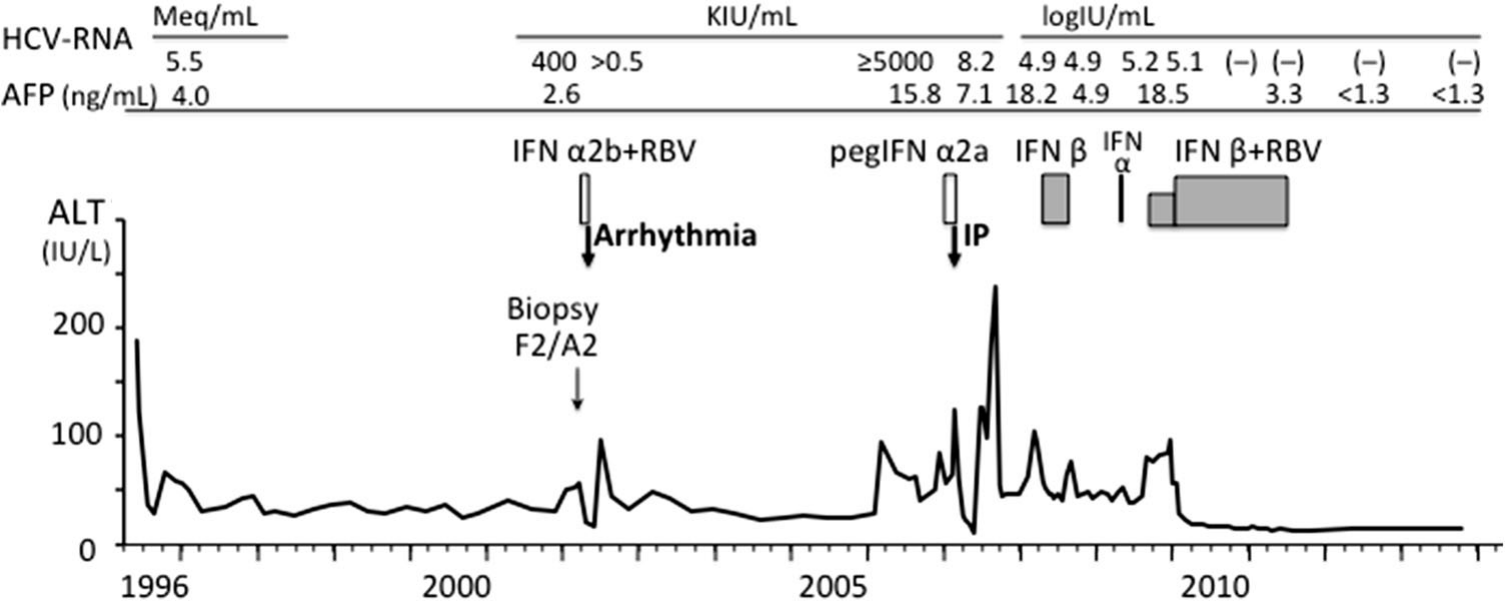
Clinical course of the patient

**Fig. 2:**
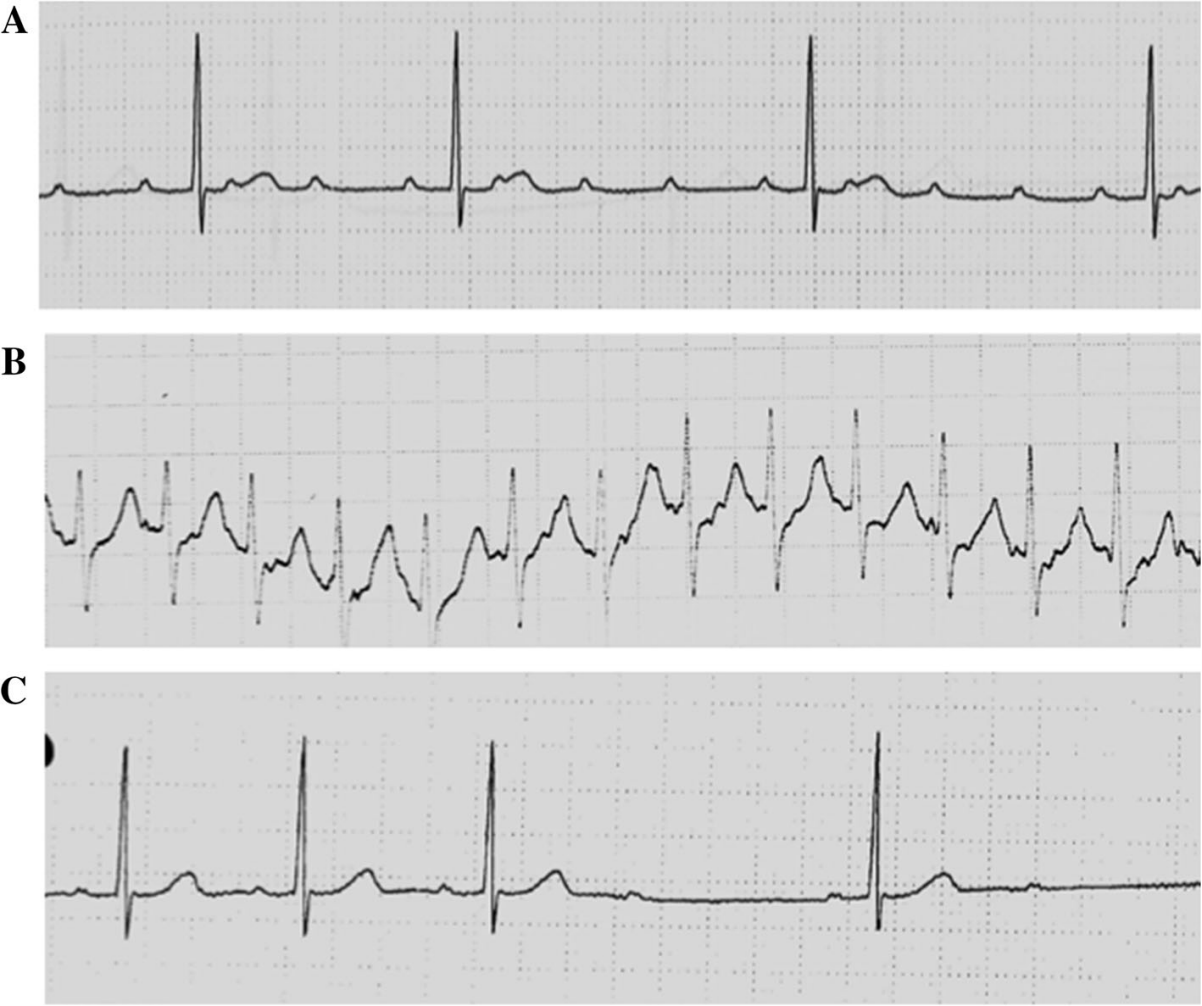
**a** Holter ECG was taken after 5 weeks of interferon-α2b plus ribavirin treatment and showed advanced atrial ventricular (AV) block. **b** Monitor ECG was taken on admission (6 weeks of treatment) and shows supraventricular tachycardia with 172 beats/min. **c** Holter ECG was taken on 4th day of admission and shows second degree AV block type 2

**Fig. 3:**
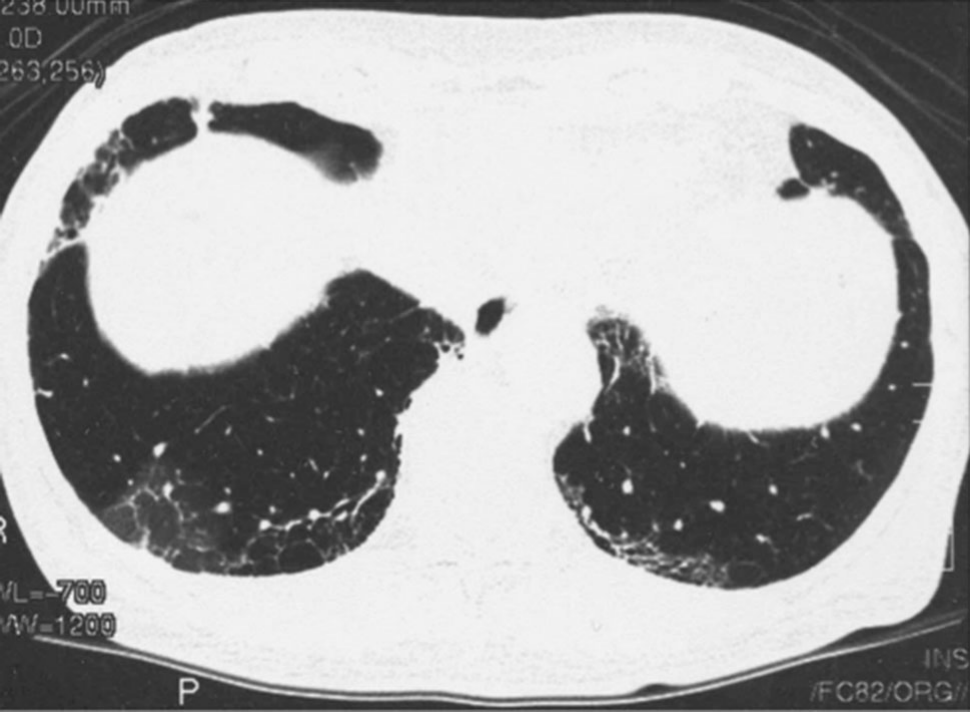
High-resolution computed tomography scan taken after 10 weeks of peginterferon-α2a treatment showing bilateral linear and lenticular interstitial opacities predominantly in the lung bases and periphery

A 56-year-old female visited our hospital for the treatment of chronic hepatitis C in 1996. She was 153 cm in height and weighed 53.0 kg. In 1984, she had received surgery for an ovarian cyst and showed abnormally high transaminase levels at that time. She had never received blood transfusions, did not consume alcohol, and had no other history of serious illness. However, she had suffered from allergic reactions to several medicines, including urticaria following chloramphenicol and tetracycline treatments, angioedema from a sulfa drug, a reddish skin reaction following a patch test for fosfomycin calcium and skin disinfection with ethanol, and suffered from liver injury after treatment with oxatomide. In 1991, she was diagnosed with chronic hepatitis C, and in 1995, she was treated with IFN-α2b for 6 months. Although a transient biochemical response was achieved, hepatitis relapsed 7 months after cessation of IFN therapy, prompting treatment with ursodeoxycholic acid (UDCA). After a subsequent visit to our hospital, laboratory examinations revealed the following biochemical levels: aspartate aminotransferase, 117 IU/l; alanine aminotransferase (ALT), 189 IU/l; albumin, 5.0 g/dl; γ-glutamyl transpeptidase, 26 IU/l; platelet count, 16.3 × 10^4^/µl; α-fetoprotein (AFP) <5 ng/ml. She carried HCV genotype 1 and had a viral load of 5.5 Meq/ml. Hepatitis B virus surface antigens and antinuclear antibodies were negative, and thyroid function was normal. Abdominal ultrasound showed no cirrhosis, no fatty liver, and no mass lesions in the liver, and the patient was diagnosed with chronic hepatitis C genotype 1, with a high viral load that was refractory to IFN monotherapy. After addition of glycyrrhizin injections to UDCA therapy, her ALT levels gradually decreased, but fluctuated between 40 and 70 IU/l (Fig. 1). In April 2002, examinations of a liver biopsy revealed chronic hepatitis (New Inuyama classification F2/A2), and combination therapy commenced with daily administration of 6 mega units (MU) IFN-α2b for 2 weeks and then three times per week, and RBV at 600 mg/day. Four weeks later, HCV-RNA levels were assessed using an Amplicor Monitor and decreased from 400 to less than 0.5 KIU/ml. However, the patient complained of an occasionally slow pulse, and a Holter electrocardiogram (ECG) after 5 weeks of treatment revealed advanced atrial ventricular (AV) block (Fig. 2a). After 6 weeks of treatment, the patient complained of palpitation attacks that persisted for more than 4 h, and ECG monitoring revealed supraventricular tachycardia of 170 beats/min (Fig. 2b). After hospital admission, her symptoms gradually subsided but sometimes relapsed, and Holter ECG showed similar supraventricular tachycardia and AV block type 2 of the second degree (Fig. 2c). Although an echocardiogram assessment showed no abnormalities, IFN treatment was considered to be the cause of arrhythmia and was stopped at the 7th week of therapy. For 4 months, arrhythmia occurred intermittently after IFN discontinuation, but it completely disappeared thereafter. She was then treated with glycyrrhizin injections and UDCA therapy. However, her ALT levels were not controlled and AFP levels gradually increased to more than 10 ng/ml. In 2007, treatment with 90-µg pegIFN-α2a every 7–10 days was started to control hepatitis. ALT and AFP levels subsequently normalized, but mild cough and sputum occurred from the 8th week of treatment. A local doctor then medicated the patient with an antitussive agent, but her symptoms deteriorated and she complained of shortness of breath at the 11th week of pegIFN-α2a treatment. Although her ALT and AFP levels remained normal and HCV-RNA levels decreased to 8.2 KIU/ml, a fine crackle was heard in her bilateral anterior lower chest, and her KL-6 levels increased to 780 U/ml. Although pulmonary function tests were normal, high-resolution computed tomography (HRCT) of the chest revealed bilateral linear and lenticular interstitial opacities, predominantly in the lung bases and periphery, indicating IP (Fig. 3). Thus, PegIFN-α2a treatments were immediately discontinued, and the patient was administered theophylline and expectorants. Gradual recovery was observed, and no respiratory symptoms appeared after 3 months, even after cessation of treatments. Although HRCT observations were almost unchanged, serum KL-6 decreased to 427 U/ml, and glycyrrhizin injections and UDCA therapy were resumed. In 2008, the patient strongly requested treatment with IFN following insufficient efficacy of other treatments and commenced with 6-MU doses of nIFN-β three times a week under careful observation. Although no respiratory symptoms occurred and AFP levels were normalized, her ALT levels were insufficiently controlled, HCV-RNA levels were unchanged (from 4.9 to 4.9 log IU/ml), and the treatment was stopped after 6 months.

In April 2009, she was administered 3 MU doses of natural IFN-α twice a week because of increases in AFP levels after discontinuation of nIFN-β therapy. Although no respiratory symptoms appeared, the patient was unable to tolerate the treatment and suffered severe neurovegetative symptoms, including fever, fatigue, nausea, and tinnitus, and she discontinued treatment after 2 weeks. In September 2009, semiweekly treatments with 6-MU nIFN-β were resumed. Laboratory data and HCV characteristics before treatment and the interleukin 28B genotype are shown in Table 1. Although AFP levels were normalized after 3 months, ALT levels and HCV-RNA load did not decrease. Therapy was continued without remarkable adverse effects, and in January 2010 (4 months after nIFN-β initiation), nIFN-β treatment was increased to three times a week with 600-mg/day RBV. Her HCV-RNA levels subsequently decreased from 5.1 to 3.4 log IU/ml at the 4th week and to 2.4 log IU/ml by the 8th week. After 24 weeks, serum HCV-RNA became undetectable, and the patient completed 72 weeks of combination therapy, which finally led to an SVR.

**Table 1:** Laboratory data before second interferon-β treatment

WBC	3100/µl	γ-GTP	33 IU/l	KL-6	485 U/ml
Hb	13.1 g/dl	TP	8.1 g/dl	SP-D	94.8 ng/ml
Hct	38.6 %	ALB	4.0 g/dl	ANA	<40
Plt	10.5 × 10^4^/µl	TC	136 mg/dl		
PT	94.0 %	UA	5.3 mg/dl	HCV	
T-Bil	0.7 mg/dl	UN	15.9 mg/dl	Genotype	1b
D-Bil	0.3 mg/dl	Cre	0.68 mg/dl	RNA load	5.2 LogIU/ml
AST	47 IU/l	Glucose	96 mg/dl	ISDR	Mixed type (0, 1, 4)
ALT	45 IU/l	HbA1c	4.9 %	Core 70AA	Wild type
LDH	164 IU/l	AFP	18.5 ng/ml	Core 91AA	Wild type
ALP	288 IU/l	PIVKA II	16 mAU/ml	IL28B rs8099917	T/T

*KL-6* normal value <500 U/ml, *SP-D* normal value <110 ng/ml

## Discussion

The patient presented here with chronic hepatitis C genotype 1b had a high viral load and suffered from two rare side effects of IFN-α treatment (arrhythmia and interstitial pneumonia), but was successfully treated with nIFN-β plus RBV therapy. It is well known that IFN treatment induces adverse effects that occasionally lead to treatment discontinuation in patients with viral hepatitis. In previous studies, rates of premature discontinuation of IFN monotherapy due to adverse effects in chronic hepatitis C patients were 4–9 % [7, 8], and they were 7–12 % for RBV combination therapy [9]. Apart from hematologic disorders, neurovegetative symptoms and depression-related syndromes are the predominant reasons for discontinuation of both IFN monotherapy and RBV combination therapy [8, 10], and dermatologic effects are also common after RBV combination therapy. Adverse cardiac events due to IFN treatments are rare among hepatitis C patients [11], and reported treatment discontinuation rates are 0.1 and 0.96 % [12, 13]. Large studies show less frequent occurrence of these events [4, 10]. However, some severe cases reportedly developed severe arrhythmia and required intensive care or cardiac pacemakers [4, 14, 15]. In contrast, the side effects of such events due to IFN-β treatment have been reported in only one patient with multiple sclerosis, who was treated with recombinant IFN-β1a [16]. Moreover, adverse cardiac effects were abolished in chronic hepatitis C patients after changing from IFN-α to nIFN-β treatments [17].

RBV is reportedly associated with bradycardia after high doses in patients with severe acute respiratory syndrome [18]. In addition, in a case study, tachycardia and syncope attacks during combined pegIFN and RBV treatment were alleviated after RBV dose reduction [19]. The patient presented here had completed 6 months of IFN-α2b monotherapy without dose reduction and had subsequently completed RBV and nIFN-β treatment without remarkable side effects. Thus, arrhythmia may have been induced by drug interactions of IFN-α2b and RBV.

Although IP is a well-known adverse effect of IFN treatments for hepatitis C patients, and sometimes leads to death [20], it occurs at a rate of only 0.3 % among Japanese patients treated with IFN monotherapy or combined RBV therapy [10, 12] and in only 0.01 % of patients in other countries [21]. To date, retreatment with IFN after the occurrence of IP has been reported in only three patients with hepatitis C. Of these, two cases changed from IFN-α2b or pegIFN-α2b to pegIFN-α2a and experienced relapse of IP [22, 23], whereas the other case changed from pegIFN-α2a to pegIFN-α2b and completed the therapy without relapse [24]. The incidence of IP due to IFN varies with IFN types and reportedly occurs in 0.29 % of cases for pegIFN-α2a, 0.12 % of cases for IFN-α2b, and 0.04 % of cases for nIFN-β [25]. In addition, the incidence of depression is lower after treatment with nIFN-β than with other types of IFN, and nIFN-β is generally tolerated in patients with a history of depression [26]. Furthermore, treatment discontinuation and/or dose reduction rates due to severe adverse effects during IFN-β treatment are generally low [5]. Thus, the Japan Society of Hepatology recommends the use of IFN-β in patients who are unable to tolerate IFN-α because of depression or other adverse effects [27]. Although the mechanisms behind improved tolerability of IFN-β remain unclear, drug concentrations after administration vary depending on the type of IFN [28], with much higher sustained serum concentrations of IFN-α2a than nIFN-β. This may reflect the production of IFN-α2a using genetic recombination and/or its requirement of subcutaneous administration. In contrast, nIFN-β is produced using human fibroblasts and is intravenously administered. Furthermore, the intracellular mechanisms of action may also differ between these IFNs. Although IFN-α and -β bind to a common type I IFN receptor, they utilize different regions of the subunit for signaling [29], producing different signaling and biological effects. In accordance, higher SVR rates have been shown with nIFN-β plus RBV therapy than with IFN-α plus RBV therapy [30], and a previous case study shows SVR following nIFN-β plus ribavirine therapy after several unsuccessful courses of IFN-α therapy with and without RBV [31].

Novel combination therapies using directly acting antivirals may become available in the near future and may produce excellent HCV eradication rates with fewer adverse effects [32]. However, some issues, such as countermeasures against drug resistance or suppressive effects on hepatocellular carcinoma development after SVR, remain unresolved. Moreover, prompt treatment is required in elderly patients and in patients with advanced liver stages to prevent hepatocellular carcinoma [27]. Hence, IFNs may remain the treatment of choice for patients with good tolerance.

In conclusion, nIFN-β plus RBV therapy is an alternative treatment option for patients who fail to tolerate the adverse effects of pegIFN-α with or without concomitant RBV therapy.

## References

[1] World Health Organization. Global Alert and Response (GAR): Hepatitis C. http://www.who.int/csr/disease/hepatitis/whocdscsrlyo2003/en/index5.html. Accessed 1 March 2014.

[2] Drafting committee for hepatitis management guidelines, the Japan Society of Hepatology. JSH Guidelines for the management of hepatitis C virus infection: a 2014 update for genotype 1. Hepatol Res. 2014; 44 Suppl S1: 59–70. 10.1111/hepr.12272 24397840

[3] Slavenburg S, Heijdra YF, Drenth JP (2010). Pneumonitis as a consequence of (peg)interferon-ribavirin combination therapy for Hepatitis C: a review of the literature. Dig Dis Sci.

[4] Fattovich G, Giustina G, Favarato S (1996). A survey of adverse events in 11,241 patients with chronic viral hepatitis treated with alfa interferon. J Hepatol.

[5] Festi D, Sandri L, Mazzela G (2004). Safety of interferon β treatment for chronic HCV hepatitis. World J Gastroenterol.

[6] Arase Y, Suzuki F, Akuta N (2010). Efficacy and safety of combination therapy of natural human interferon beta and ribavirin in chronic hepatitis C patients with genotype 1b and high virus load. Intern Med.

[7] Poynard T, Leroy V, Cohard M (1996). Meta-analysis of interferon randomized trials in the treatment of viral hepatitis C: effects of dose and duration. Hepatology.

[8] Iino S (1993). High dose interferon treatment in chronic hepatitis C. Gut.

[9] Fried MW, Shiffman ML, Reddy R (2002). Peginterferon alfa-2a plus ribavirin for chronic hepatitis C virus infection. N Engl J Med.

[10] Ogawa E, Furusyo N, Kajiwara E (2012). Evaluation of the adverse effect of premature discontinuation of pegylated interferon α-2b and ribavirin treatment for chronic hepatitis C virus infection: results from Kyushu University liver disease study. J Gastroenterol Hepatol.

[11] Fried MW (2002). Side effects of therapy of hepatitis C and their management. Hepatology.

[12] Okanoue T, Sakamoto S, Itoh Y (1996). Side effect of high-dose interferon therapy for chronic hepatitis C. J Hepatol.

[13] Teragawa H, Hondo T, Amanao H (1996). Adverse effects of interferon on the cardiovascular system in patients with chronic hepatitis C. Jpn Heart J.

[14] Teragawa H, Hondo T, Amano H (1996). Cardiogenic shock following recombinant alpha-2b interferon therapy for chronic hepatitis C. A case report. Jpn Heart J.

[15] Rechcinski T, Matusik D, Rudzunski T (2007). Cardiotoxic properties of interferon: aggravation of atrio-ventricular block during treatment of chronic hepatitis C with peginterferon–a case report. Pol Arch Med Wewn.

[16] Kastalli S, Aïdli SE, Moudrali S (2012). Cardiac arrhythmia induced by interferon beta-1a. Fundam Clin Pharmacol.

[17] Sasaki M, Sata M, Suzuki H (1998). A case of chronic hepatitis C with sinus bradycardia during IFN therapy. Kurume Med J.

[18] Muller MP, Dresser L, Raboud J (2007). Adverse events associated with high-dose ribavirin: evidence from the Toronto outbreak of severe acute respiratory syndrome. Pharmacotherapy.

[19] El-Atrebi K, El-Bassyouni HT (2009). Management of rare side effects of peginterferon and ribavirin therapy during hepatitis C treatment: a case report. Cases J.

[20] Slavenburg S, Heijdra YF, Drenth JPH (2010). Pneumonitis as a consequence of (peg)interferon-ribavirin combination therapy for hepatitis C: a review of the literature. Dig Dis Sci.

[21] Solsky J, Liu J, Peng M (2009). Rate of interstitial pneumonitis among hepatitis C-infected patients treated with peginterferon. J Hepatol.

[22] Kumar KS, Russo MW, Borczuk AC (2002). Significant pulmonary toxicity associated with interferon and ribavirin therapy for hepatitis C. Am J Gastroenterol.

[23] Renou C, Germain S, Harafa A (2005). Interstitial pneumonia recurrence during chronic hepatitis C treatment. Am J Gastroenterol.

[24] Shimada M, Yoshida S, Korneck M (2010). A change of peginterferon may permit continuation of antiviral therapy in hepatitis C virus-infected patients with interstitial pneumonitis. Liver Int.

[25] Pharmaceutical and Safety Bureau, Ministry of Japanese Health, Labour and Welfare. Pharmaceutical and medical devices safety information 2008;250.

[26] Arase Y, Suzuki Y, Suzuki F (2011). Efficacy and safety of combination therapy of natural human interferon beta and ribavirin in chronic hepatitis C patients. Int Med.

[27] Editors in drafting committee for hepatitis management guidelines: the Japan Society of Hepatology. Guidelines for the management of hepatitis C virus infection: First edition, May 2012, the Japan Society of Hepatology. Hepatol Res. 2013; 43:1–34. 10.1111/hepr.12020 23332085

[28] Furue H, Kobayashi H, Komuro T (1984). Pharmacokinetics of human interferos. Jpn Cancer Chemother.

[29] Domanski P, Nadeau OW, Platanias LC (1998). Differential use of the β_L_ subunit of the type 1 interferon (IFN) receptor determines signaling specificity for IFNα2 and IFNβ. J Biol Chem.

[30] Enomoto M, Tamori A, Kawada N (2006). IFN-β plus ribavirin for patients with hepatitis C virus genotype 1: a randomized pilot trial. Gut.

[31] Kanda T, Nakamoto S, Arai M (2013). Natural interferon-beta plus ribavirin therapy led to sustained virological response after seven unsuccessful courses of anti-viral treatment in a chronic hepatitis C patient. Clin J Gastroenterol.

[32] Asselah T, Marcellin P (2013). IFN free therapy with direct acting antivirals for HCV. Liver Int.

